# Treatment of ruptured intracranial dissecting aneurysms in Hong Kong

**DOI:** 10.4103/2152-7806.74145

**Published:** 2010-12-22

**Authors:** George Kwok Chu Wong, Hoi Bun Tang, Wai Sang Poon, Simon Chun Ho Yu

**Affiliations:** Division of Neurosurgery, Prince of Wales Hospital, The Chinese University of Hong Kong, Shatin, Hong Kong; 1 Department of Imaging and Interventional Radiology, Prince of Wales Hospital, The Chinese University of Hong Kong, Shatin, Hong Kong

**Keywords:** Cerebral aneurysm, dissection, embolization, stent, subarachnoid hemorrhage

## Abstract

**Background:**

Data suggests that hemorrhagic presentations occur in 20% of internal carotid artery dissections and 50% of vertebral artery dissections. A Finnish study has reported favorable outcomes in only 32% of patients.

We aimed to review the epidemiology and management outcomes in a Chinese population.

**Methods:**

We reviewed the aneurysmal subarachnoid hemorrhage registry of patients who presented with intracranial dissecting aneurysms at a neurosurgical center in Hong Kong over a five-year period.

**Results:**

A total of 23 patients with intracranial dissecting aneurysms were identified, accounting for 8% of all spontaneous subarachnoid hemorrhage patients. Forty-eight percent of the patients identified were treated by main trunk occlusion and 39% were treated by embolization or stent-assisted embolization or stent alone. Thirteen percent were managed by craniotomy and trapping or wrapping. Favorable outcomes at six months were achieved in 67%.

**Conclusions:**

Patients with intracranial dissecting aneurysms account for a significant proportion of the cases of spontaneous subarachnoid hemorrhage in our population. Carefully selected endovascular and microsurgical treatments can lead to management outcomes similar to patients with saccular aneurysms.

## INTRODUCTION

The data suggests that hemorrhagic presentations occur in 20% of internal carotid artery dissections and 50% of vertebral artery dissections.[15] A rebleeding rate of more than 40% within the first 24 h has been reported.[7] The absence of an external elastic membrane and the presence of thin muscular and adventitial layers make intracranial arteries potentially prone to subadventitial dissection and subsequent subarachnoid hemorrhage. Subarachnoid hemorrhage occurs when the dissecting lesion is between the media and either the adventitia or the transmural.[3,6,13] The imaging findings can be dilatation (fusiform or rosette), coexistence of stenosis and post-lesional dilatation (string sign), and double lumen images (intimal flap).[10]

In contrast to the high percentage of favorable outcomes in nonhemorrhagic patients, the Finnish study reported favorable outcomes in only a third of patients.[6] This concurs with their review of case series occurring before 2000.[6] With the subsequent evolution of endovascular embolization and stenting, more promising management results have been reported.[1,4,8,9,11–14] Epidemiological data for Chinese patients are limited. We therefore aimed to review the epidemiology and management outcomes in a Chinese population.

## MATERIALS AND METHODS

We reviewed the aneurysmal subarachnoid hemorrhage registry of patients who presented with intracranial dissecting aneurysms at a neurosurgical center in Hong Kong over a five-year period. Because our institute introduced multi-slice computed tomographic angiography and flat panel biplane digital subtraction angiography five years ago, we decided to review the data for this five-year period. Patients were identified by reviewing the institutional database of aneurysmal subarachnoid hemorrhage patients.

The patient’s condition on admission was evaluated according to the Glasgow coma scale (GCS) and the world federation of neurological surgeons grading (WFNS). Patient histories were reviewed for hypertension, diabetes, medical history, and family history. Clinical outcomes were determined using a modified Rankin score (mRS), with favorable outcomes defined as 0-2. Follow-up angiographies were performed at six months and eighteen months to detect any signs of recurrence.

Endovascular treatment was chosen as the first option and was performed depending on the patient’s status,[2] the location of the dissection, anatomy of related vessels, and possible collateral circulation. Endovascular treatments were performed directly after the diagnostic angiography during the acute phase. The interventions were usually performed under general anesthesia and balloon occlusion tests with angiographic analysis were performed when needed. The evaluations of collateral circulation were usually based on meticulous arterial anatomical analyses (reconstitution of the distal arterial territory) and venous delay analysis (less than 2 sec delay) conducted under general anesthesia, rather than relying on patient’s cooperation during the acute phase of subarachnoid hemorrhage. Patients were considered for trapping of the dissecting aneurysm if the lesion did not incorporate a branch, such as the posterior inferior cerebellar artery. This was accomplished with coil embolization using Guglielmi detachable coils (GDC, Boston Scientific, Fermont, CA) or Matrix2 coils (Boston Scientific, Fermont, CA). An intravenous bolus of heparin at 50 units per kg was given before embolization and low-molecular-weight heparin was given for the subsequent two days (fraxiparine 0.3-0.4 ml twice daily). After occlusion, patients were monitored in the neurosurgical high dependency unit. If the dissecting segment incorporated an important branch or represented the sole supply to the territory, stent alone or stent-assisted embolization would be considered. Double antiplatelets (plavix 75 mg daily and aspirin 160 mg daily) were then prescribed for three months, followed by aspirin 160 mg daily for another three months.

Statistical analysis was carried out using SPSS for Windows Version 15.0. A Fisher exact test and a Chi-square test were carried out as appropriate. Statistical significance was taken as *P*-value <0.05.

## RESULTS

Twenty-three patients with intracranial dissecting aneurysms were identified over a five-year period [Table 1]. They accounted for 8% of all spontaneous subarachnoid hemorrhage patients. Age (mean ± SD) was 56.3 ± 10.1 years. Thirteen were females and ten were males. Twelve (52%) had hypertension and three (13%) had diabetes mellitus. One patient had systemic vasculitis at the time of presentation. One patient had ischemic heart disease and one patient had hyperlipidemia. Median GCS on admission was 13, with interquartile range between 4 and 15. Twelve (52%) were poor grade (WFNS Grade 1-3). Fifteen (65%) dissecting aneurysms occurred in the intracranial vertebral artery; four (17%) occurred in the intracranial internal carotid artery; three (13%) occurred in the anterior cerebral artery; and one (4%) occurred in the middle cerebral artery.

**Table 1:** Patient profile

Age	Sex	Admission WFNS	Site of dissecting aneurysm	Procedure	Complications	Follow up (months)	GOS during last follow up
50	F	1	Right vertebral artery	Main trunk occlusion		53	4
72	F	1	Right vertebral artery	Main trunk occlusion		52	4
49	F	2	Right vertebral artery	Stent-assisted embolization		44	5
57	M	5	Right vertebral artery	Main trunk occlusion	Midbrain hemorrhage after Ilb/IIIa receptor antagonist for PICA occlusion	1	1
47	F	5	Right vertebral artery	Aneurysm embolization		40	4
79	F	1	Right vertebral artery	Aneurysm embolization		42	3
54	M	1	Right vertebral artery	Aneurysm embolization		37	3
57	M	1	Right vertebral artery	Main trunk occlusion		24	3
52	F	5	Left anterior cerebral artery	Stent-assisted embolization		27	5
59	F	4	Left vertebral artery	Main trunk occlusion		30	5
67	F	5	Right vertebral artery	Main trunk occlusion		15	5
52	M	4	Left vertebral artery	Main trunk occlusion		12	4
56	F	4	Right anterior cerebral artery	Main trunk occlusion		11	3
58	M	1	Left vertebral artery	Flow-diverting stent		8	5
51	M	4	Right anterior cerebral artery	Main trunk occlusion	Aneurysm recanalization and rebleed	8	3

WFNS: World federation of neurological surgeons grading; GOS: Glasgow outcome scale

Eleven (48%) patients were treated with main trunk occlusion through endovascular coiling; five (22%) patients were treated with embolization alone; two (9%) patients were treated with stent-assisted embolization; one patient had covered stent placement (JOSTENT, Abbott Vascular, Illinois); one patient was treated with a flow diverter (Pipeline Embolization Device [PED], eV3 Neurovascular, Irvine CA); and one patient had the aneurysm spontaneously thrombosed during embolization.

Three (13%) patients were treated with microsurgery. One patient was an 80-year-old woman who presented with WFNS Grade IV subarachnoid hemorrhage. An angiogram showed a 2 mm outpouch in the proximal middle cerebral artery. Because of the size of aneurysm, she received microsurgical exploration. Intraoperative findings confirmed dissection of the temporal branch of the middle cerebral artery, which was wrapped. She subsequently remained dependent. The other two patients had dorsal ophthalmic segment internal carotid artery blister aneurysms, for which one received surgical trapping and the other microsurgical clipping. They remained well on clinical follow up.

The follow-up period ranged from 1 to 53 months, with a mean of 28 months. Fourteen (67%) patients had a favorable neurological outcome. There were two (9%) deaths.

Two (9%) patients suffered from recurrent subarachnoid hemorrhage. One patient had proximal anterior cerebral artery dissecting aneurysm with coil embolization of the aneurysm together with anterior cerebral artery (main trunk occlusion). He developed recurrent hemorrhage presenting with decreased level of consciousness one month afterwards. An angiogram showed recanalization of the aneurysm, and a repeat embolization was performed. He subsequently remained dependent. Another patient had the dorsal ophthalmic segment internal carotid artery blister aneurysm spontaneously thrombosed during embolization. She suffered from recurrent hemorrhage with neurological deterioration two months afterwards and died during the same admission. Otherwise, there were no other recurrences detected during follow-up angiographies.

There were two (9%) procedural complications. One patient had a dissecting vertebral artery aneurysm just after the origin of the posterior inferior cerebellar artery. He received coil embolization of the aneurysm, together with main trunk occlusion of the vertebral artery, with the aim of preservation of flow to the posterior inferior cerebellar artery. However, sluggish flow to the posterior inferior cerebellar artery was noted at the end of the procedure. Intra-arterial abciximab (glycoprotein IIb/IIIa inhibitor) was given to restore flow to the posterior inferior cerebellar artery. He was then noted as having dilated pupils. Urgent computed tomography of the brain showed extensive midbrain hemorrhage and he subsequently died during the same admission. Another patient, who had covered stent insertion for the vertebral artery dissecting aneurysm between the origin of the posterior inferior cerebellar artery and the vertebrobasilar junction, developed cerebellar infract post-operatively. Total occlusion of the vertebral artery with sluggish flow to the posterior inferior cerebellar artery was found in the follow-up angiography.

## CASE REPORTS

### Case 1

**Figure 1:**
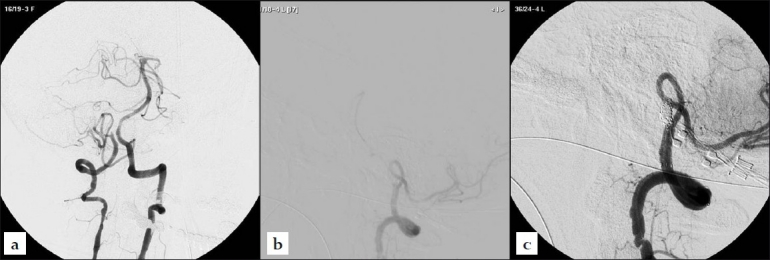
(a) Simultaneous bilateral vertebral artery injections showing apparent occlusion of the right vertebral artery just distal to the origin of the posterior inferior cerebellar artery with a 2 mm remnant aneurysmal stump compatible with dissection; (b) Selective injection of the right vertebral artery showing a thin line of contrast passing the distal to the stump compatible with string sign; (c) Post-stent-assisted embolization DSA showing occlusion of the dissecting segment of the right vertebral artery with preservation of the posterior inferior cerebellar artery.

A 49-year-old female presented to us after sudden collapse, with GCS 8/15. A CT brain scan showed extensive diffuse subarachnoid hemorrhage over the basal cisterns and hydrocephalus. She was intubated and an external ventricular drain was inserted to relieve the hydrocephalus. She had complications of neurogenic pulmonary edema and thrombocytopenia. Digital subtraction angiography on the next day showed features of dissecting aneurysm of the right vertebral artery, just after the origin of the posterior inferior cerebellar artery [Figure 1a and b]. A Neuroform2 stent (Boston Scientific, Fermont, CA) was inserted to protect the posterior inferior cerebellar artery and GDC occlusion of the remnant of the right vertebral artery was performed using sequential technique [Figure 1c]. She recovered completely at three months and follow-up angiography showed stable occlusion with preservation of flow to the posterior inferior cerebellar artery.

### Case 2

**Figure 2:**
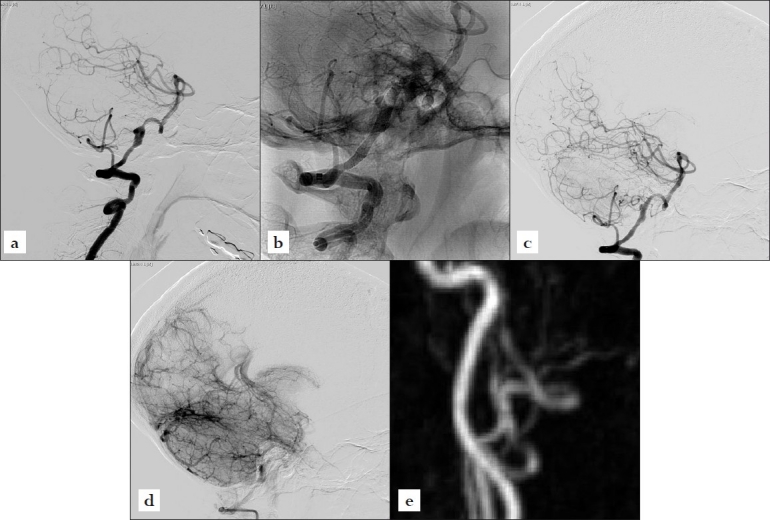
(a) Vertebral artery angiography showing a dissecting aneurysm distal to the origin of the posterior inferior cerebellar artery; (b) Non-subtracted angiography showing the two stents *in-situ*; (c) Post-PED insertion angiography in arterial phase showing partial aneurysm thrombosis; (d) Post-PED insertion angiography in the venous phase showing contrast stasis; (e) Six-month follow up MR angiography showing complete occlusion of the aneurysm and preservation of flow to the left vertebral artery.

A 59-year-old female presented with sudden collapse, with GCS 5/15, to a local hospital while travelling. A CT brain scan showed diffuse subarachnoid hemorrhage over the basal cisterns. She recovered in the following two weeks and was transferred back to us for further management. Digital subtraction angiography showed a dominant left vertebral artery dissecting aneurysm distal to the origin of the posterior inferior cerebellar artery [Figure 2a]. In view of the delay, a decision was made for flow diverters to occlude the aneurysm and preserve the main trunk. She was given a loading dose of plavix 300 mg and aspirin 160 mg in the morning of the procedure. After an intravenous dose of heparin 3500 units, two pipeline embolization devices (PED) were deployed to cover the whole diseased segment [Figure 2b]. Check DSA showed immediate partial thrombosis of the aneurysm and contrast stasis [Figure 2c and d]. She was given plavix 75 mg daily for six weeks and aspirin 160 mg daily for six months. Six month angiography showed complete occlusion of the aneurysm with preservation of the left vertebral artery [Figure 2e].

## DISCUSSION

Because dissecting aneurysms have a pseudoaneurysm nature, direct microsurgical clipping is usually not feasible. Among the different causes of morbidity and mortality, or reasons for surgical failure, some authors suggest that thrombosis of the tiny perforating branches during surgical trapping contributes to the poor outcomes.[10] With the availability of endovascular treatments and the advantage of intraprocedural anticoagulation, endovascular main trunk occlusion has become the primary treatment for most patients.[9,14] In these two recent series of endovascular occlusions for hemorrhagic vertebrobasilar dissection, 22 out of 33 patients (67%) reported favorable outcomes. If the circulation is judged not to be suitable for simple trapping, bypass procedure or, more recently, stenting is recommended.

A Pubmed review of treatments of hemorrhagic dissecting intracranial aneurysms, from January 2000 to December 2009, showed four case series that focused on stent alone treatment or stent-assisted embolization and one case series on covered stent.[1,5,8,11,12] In the first four case series mentioned, all 29 patients had hemorrhagic dissecting vertebrobasilar artery aneurysms on presentation. Sixteen patients underwent stent alone insertion and thirteen patients underwent stent-assisted coil embolization. Favorable outcomes were achieved in 25 (86%) patients. There were no significant differences in outcome between stent-assisted embolization and stent alone treatment (*P*=0.299). Angiographic improvement of incompletely occluded aneurysms was noted in 77% patients who underwent angiographic follow-up. There was one (3%) rebleeding up to the last follow up. Complete occlusion at the last follow up was achieved in 77% of cases with stent-assisted embolization and 44% of cases with stent alone treatment, *P*=0.130.

Theoretically, a covered stent graft for a segment without important branches is the simplest solution to the problem. The group from West China Hospital reported the application of covered stent grafts for five patients with hemorrhagic intracranial vertebral artery dissecting aneurysms.[4] There was one unsuccessful navigation and placement, with no periprocedural complications. All the patients received angiographic follow up 6 to 14 months later. We also had one patient who received a similar covered stent insertion and an anticoagulation/antiplatelet regimen. However, this resulted in total thrombotic occlusion of the segment. The JOSTENT used by the West China Hospital and our group is a stainless steel stent with a PTFE membrane placed between two layers of stent struts. The large amount of foreign material is expected to be more thrombogenic than the thin nitinol struts employed in other stents designed for stent-assisted embolization. Flow diverter, pipeline embolization device (ev3 neurovascular, Irving, CA), was successfully deployed in one of our recent patients, are expected to be a good compromise between the two. Safety profiles, in terms of thromboembolic complications and side branch preservations, have been confirmed in a recent case series.[5]

One limitation of the current study is the small sample size from a single neurosurgical center. Multi-center registry is useful to confirm the finding.

## CONCLUSIONS

Dissecting intracranial aneurysms account for a significant proportion of the patients with spontaneous subarachnoid hemorrhage in our population. Carefully selected endovascular and microsurgical treatments can lead to management outcomes similar to patients with saccular aneurysms.

## References

[1] Ahn JY, Chung SS, Lee BH, Kim SH, Yoon PH, Joo JY (2005). Treatment of spontaneous arterial dissections with stent placement for preservation of the parent artery. Acta Neurochir (Wien).

[2] Ahn JY, Han IB, Kim TG, Yoon PH, Lee YJ, Lee BH (2006). Endovascular treatment of intracranial vertebral artery dissections with stent placement or stent-assisted coiling. Am J Neuroradiol.

[3] Farrell M, Gilbert JJ, Kaufmann (1985). Fatal intracranial arterial dissection: Clinical pathological correlation. J Neurol Neurosurg Psychiatry.

[4] He M, Zhang H, Lei D, Mao BY, You C, Xie XD (2009). Application of covered stent grafts for intracranial vertebral artery dissecting aneurysms. J Neurosurg.

[5] Lylyk P, Miranda C, Ceratto R, Ferrario A, Scrivano E, Luna HR (2009). Curative endovascular reconstruction of cerebral aneurysms with the Pipeline Embolization Device: the Buenos Aires experience. Neurosurgery.

[6] Metso TM, Metso AJ, Helenius J, Haapaniemi E, Salonen O, Porras M (2007). Prognosis and safety of anticoagulation in intracranial artery dissections in adults. Stroke.

[7] Mizutani T, Kojima H, Asamoto S, Milky Y (2001). Pathological mechanism and three-dimensional structure of cerebral dissecting aneurysms. J Neurosurg.

[8] Park SI, Kim BM, Kim DI, Shin YS, Suh SH, Chung EC (2009). Clinical and angiographic follow-up of stent-only therapy for acute intracranial vertebrobasilar dissecting aneurysms. Am J Neuroradiol.

[9] Rabinov JD, Hellinger FR, Morris PP, Oglivy CS, Putman CM (2003). Endovascular management of vertebrobasilar dissecting aneurysms. Am J Neuroradiol.

[10] Santos-Franco JA, Zenteno M, Lee A (2008). Dissecting aneurysms of the vertebrobasilar system. A comprehensive review on natural history and treatment options. Neurosurg Rev.

[11] Suh SH, Kim BM, Park SI, Kim DI, Shin YS, Kim EJ (2009). Stent-assisted coil embolization followed by a stent-within-a-stent technique for ruptured dissecting aneurysms of the intracranial vertebrobasilar artery. J Neurosurg.

[12] Wakhloo AK, Mandell J, Gounis MJ, Brooks C, Linfante I, Winer J (2008). Stent-assisted reconstructive endovascular repair of cranial fusiform atherosclerotic and dissecting aneurysms: Long-term clinical and angiographic follow-up. Stroke.

[13] Yoon W, Seo JJ, Kim TS, Do HM, Jayaraman MV, Marks MP (2007). Dissecting of the V4 segment of the vertebral artery: Clinicoradiological manifestations and endovascular treatment. Eur Radiol.

[14] Zhao WY, Krings T, Alvarez H, Ozanne A, Holmin S, Lasjaunias P (2007). Management of spontaneous haemorrhagic intracranial vertebrobasilar dissection: Review of 21 consecutive cases. Acta Neurochir (Wien).

[15] Zweifler R, Weir B, Wolff PA (2004). Stroke: Pathophysiology, diagnosis and management.

